# *AMMECR1*: a single point mutation causes developmental delay, midface hypoplasia and elliptocytosis

**DOI:** 10.1136/jmedgenet-2016-104100

**Published:** 2016-11-03

**Authors:** Gaia Andreoletti, Eleanor G Seaby, Jennifer M Dewing, Ita O'Kelly, Katherine Lachlan, Rodney D Gilbert, Sarah Ennis

**Affiliations:** 1 Human Genetics & Genomic Medicine, University of Southampton, Duthie Building (Mailpoint 808), Southampton General Hospital, Southampton, UK; 2 Centre for Human Development, Stem Cells and Regeneration HDH, University of Southampton, IDS Building, Southampton General Hospital, Southampton, UK; 3 Wessex Clinical Genetics Service, University Hospital Southampton NHS Foundation Trust, Princess Anne Hospital, Southampton, UK; 4 Wessex Regional Paediatric Nephro-Urology Service, Southampton Children's Hospital, Southampton, UK; 5 Faculty of Medicine, University of Southampton, Southampton, UK

**Keywords:** Clinical genetics, Complex traits, Diagnosis, Molecular genetics

## Abstract

**Background:**

Deletions in the Xq22.3–Xq23 region, inclusive of *COL4A5*, have been associated with a contiguous gene deletion syndrome characterised by Alport syndrome with intellectual disability (Mental retardation), Midface hypoplasia and Elliptocytosis (AMME). The extrarenal biological and clinical significance of neighbouring genes to the Alport locus has been largely speculative. We sought to discover a genetic cause for two half-brothers presenting with nephrocalcinosis, early speech and language delay and midface hypoplasia with submucous cleft palate and bifid uvula.

**Methods:**

Whole exome sequencing was undertaken on maternal half-siblings. In-house genomic analysis included extraction of all shared variants on the X chromosome in keeping with X-linked inheritance. Patient-specific mutants were transfected into three cell lines and microscopically visualised to assess the nuclear expression pattern of the mutant protein.

**Results:**

In the affected half-brothers, we identified a hemizygous novel non-synonymous variant of unknown significance in *AMMECR1* (c.G530A; p.G177D), a gene residing in the AMME disease locus. Transfected cell lines with the p.G177D mutation showed aberrant nuclear localisation patterns when compared with the wild type. Blood films revealed the presence of elliptocytes in the older brother.

**Conclusions:**

Our study shows that a single missense mutation in *AMMECR1* causes a phenotype of midface hypoplasia, mild intellectual disability and the presence of elliptocytes, previously reported as part of a contiguous gene deletion syndrome. Functional analysis confirms mutant-specific protein dysfunction. We conclude that *AMMECR1* is a critical gene in the pathogenesis of AMME, causing midface hypoplasia and elliptocytosis and contributing to early speech and language delay, infantile hypotonia and hearing loss, and may play a role in dysmorphism, nephrocalcinosis and submucous cleft palate.

## Introduction

The chromosomal region Xq22.3–Xq23 encompasses the entire *COL4A5* gene and its adjacent genes extending towards the telomere: *GUCY2F*, *NXT2*, *KCNE1L*, *ACSL4*, *TMEM164*, *MIR3978*, *AMMECR1*, *SNORD96B*, *RGAG1*, *TDGF3, CHRDL1, PAK3* and *DCX*. Deletions in this region have been associated with a contiguous gene deletion syndrome characterised by Alport syndrome with intellectual disability, midface hypoplasia and elliptocytosis (AMME (MIM: 300194)). The first report of AMME was by Jonsson *et al*^1^ who reported a microdeletion spanning from *COL4A6* (upstream of *COL4A5*) to *TDGF3* in two affected brothers. *COL4A5*, the gene underlying X-linked Alport syndrome (AS (MIM: 301050)), maps to Xq22.3 and is well known to cause renal failure, sensorineural hearing loss and ocular abnormalities.^2^ However, mutations in *COL4A5* do not account for the additional manifestations seen in AMME; this suggests that neighbouring genes of the Alport locus contribute to the rest of the phenotype. Until recently, the significance of these adjacent genes has been largely speculative.

We report on two maternal half-brothers, presenting with nephrocalcinosis and midface hypoplasia. The older sibling also has deafness and elliptocytosis. By application of next-generation sequencing, we identified a novel point mutation in *AMMECR1* (c.G530A; p.G177D) present in the half-siblings and carried in the mother. We show that this variant alone causes midface hypoplasia, speech and language delay and elliptocytosis, emphasising the critical importance of *AMMECR1* in the pathogenesis of AMME.

## Methods

**Figure 1:**
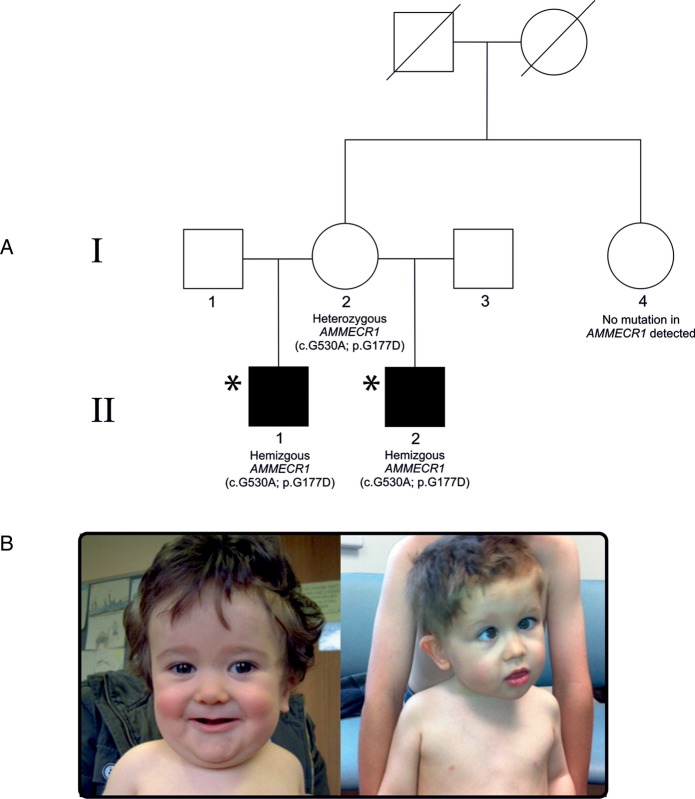
(A) Family pedigree for proband II(1), and maternal half-brother II(2) with genotype information for all individuals tested. Affected individuals are in black. The maternal parents were phenotypically normal. Asterisk defines individuals who underwent whole exome sequencing. (B) Photographs of proband II(1) (taken at 11 months) (left) and proband II(2) (taken at 2 years and 5 months) (right).

Two maternal Caucasian half-siblings presenting with overlapping phenotypes (figure 1) were ascertained through the paediatric nephrology service at University Hospital Southampton.

### Proband II(1)

Following a pregnancy complicated by extreme hyperemesis, proband II(1) was delivered by elective caesarean section for breech presentation weighing 3.59 kg (just above 50th centile). Antenatal screening showed evidence of fetal nuchal oedema, bilateral talipes, short femurs and decreased liquor. An amniocentesis showed a normal 46,XY karyotype. Shortly after birth, proband II(1) was noted to have hypotonia and poor feeding. Methylation testing of *SNRPN* exon 1 in the PWS/AS critical region was normal. Apparently short femora, bilateral talipes, widely spaced nipples, a flat facial profile with a flat nasal bridge, downslanting palpebral fissures, thin upper lip, micrognathia, short neck, folded upper helix to the ears, fifth finger clinodactyly and additional right nipple were noted. He had surgical correction for a submucous cleft palate and bifid uvula. An abdominal ultrasound scan showed possible nephrocalcinosis. An ultrasound of the head was normal.

Aged 2 months, he underwent bilateral percutaneous achilles tenotomies. His length was just above the 0.4th centile, weight on the 2nd centile and with a disproportionately large head circumference on the 50th centile. Bilateral nephrocalcinosis was confirmed on a repeat ultrasound scan. At seven and a half months, following application of the Alberta Infant Motor Scale,^3^ he was assessed to have mild-to-moderate delay in motor and communication skills. Diagnoses of Stickler syndrome and chondrodysplasia punctata were considered. Very long chain fatty acid tests were normal. Vision and fundal examination were normal on formal ophthalmic examination. Skeletal surveys performed just before his first birthday, and repeated when aged 4, reported no skeletal abnormality and specifically no epiphysial stippling. He was discharged from physiotherapy, occupational therapy and speech and language therapy before his second birthday, having made improvements in his gross motor development and feeding following cleft palate repair. Aged 23 months, he had a normal brain MRI with no evidence of hypothalamic or pituitary abnormality, ruling out a midline defect. He did not get his first teeth until he was 21 months old.

Aged 3, his short stature (0.4th centile) was calculated to be within the target centile range for his parents' heights. His head remained disproportionately large (50th centile) with weight on the 2nd centile. Aged 4, he was noted to have mild facial dysmorphology consisting of a flat midface with a large, broad forehead and small mouth with crowded teeth. There were bulbous tips to his toes and marked lumbar lordosis without scoliosis. There was a normal sitting height to leg length ratio. He had started mainstream school; however, there was still parental concern regarding slow progress and poor concentration. He had repeatedly elevated urine calcium:creatinine ratios, but a persistently normal plasma calcium concentration. Further ultrasound scans corroborated previous findings of nephrocalcinosis. At his most recent review, aged 11, proband II(1) remained in mainstream school. His facial dysmorphic features were much less apparent than observed previously, and his growth parameters were unchanged. He had bilateral mixed hearing loss requiring hearing aids; sensorineural hearing loss was first detected at age 3, despite a normal audiogram 1 year previously. His bone conduction threshold was 35–45 dB bilaterally, whereas air conduction showed a threshold of 45–50 dB bilaterally. He had also begun to suffer from migraines. He had mild joint hypermobility but much improved hypotonia. He had persistent nephrocalcinosis with intermittent hypercalciuria.

### Younger maternal half-brother II(2)

Proband II(2) was born by emergency caesarean section for failure to progress at 39 weeks’ gestation weighing 3.40 kg. His pregnancy was complicated by polyhydramnios and hyperemesis similar to his older half-brother. Otherwise, antenatal screening was normal. He had congenital dysplasia of the hips treated with a Pavlik harness. Retrospective review of early radiographs showed no evidence of epiphysial stippling. Shortly after birth, it was noted that he shared dysmorphic facial features with his half-brother. He also had a submucous cleft palate, bifid uvula and short stature. Aged 1, he underwent a renal ultrasound scan, which revealed bilateral renal dysplasia. The right kidney measured 5.4 cm and the left was 6 cm. There was early nephrocalcinosis. A simple cyst was seen in the midpole of the left kidney. He did not have the significant infantile hypotonia observed in his brother and met his motor milestones appropriately. However, he had more significant speech and language delay, with no words at 2 years of age. Aged 3, he had made progress and was putting two words together. Similar to his brother, his growth parameters followed below the 0.4th centile for height, with weight between the 2nd and 9th centile and head circumference between the 25th and 50th centiles. He had joint hypermobility, right esotropia and viral-induced wheeze. Aged 4, an audiogram showed air conduction thresholds of 20 dB bilaterally, just within the normal range. Bone conduction was tested only on the right with a threshold of 10 dB across the frequency range. This audiogram showed deterioration from one previously. Aged 5, proband II(2) was developing well and displayed a degree of developmental catch up with improved speech and language skills; he attended a normal school. His facial dysmorphology was also less apparent.

### Mother I(2)

Their mother was of normal intelligence. Renal ultrasound scan performed when she was 34 years was normal. She had normal renal function and there was no evidence of hypercalciuria. The maternal grandparents were deceased but were reported as showing none of the abnormal phenotypic features of the probands. There were no other male relatives.

### Clinical genetic testing

An array comparative genomic hybridisation (CGH) was performed on proband II(1) and showed no copy number variations (CNVs; Agilent 44 K oligo array-CGH). A multidisciplinary discussion informed selection of a gene candidate, *OCRL1*, an X-linked gene involved in Lowe oculocerebrorenal syndrome, Fanconi syndrome, intellectual disability and nephrocalcinosis (MIM: 309000).^4^ Sanger sequencing of *OCRL1* revealed no mutations in either sibling. As a result, the half-siblings were referred to the Southampton Genomic Informatics Group for whole exome sequencing.

### DNA extraction

Genomic DNA (gDNA) was extracted from peripheral venous blood samples collected in an ethylenediaminetetraacetic acid tube. DNA concentration was estimated using the Qubit 2.0 Fluorometer. The average gDNA yield obtained was 150 µg/mL. Approximately, 20 µg of gDNA was used per patient for next-generation sequencing.

### Whole exome sequencing data generation and data analysis

Whole exome sequencing was performed on two maternal half-siblings using the Agilent SureSelect Human all Exon 51 Mb V.5 capture kit. As previously described,^5^ ^6^ FASTQ raw data generated from Illumina paired-end sequencing were aligned against the human reference genome (hg19) using Novoalign (novoalign/2.08.02). SAMtools mpileup tool (samtools/0.1.19)^7^ was used to detect the variation from the mapping information to call SNPs and short INDELs from the alignment file. ANNOVAR (annovar/21 February 2013)^8^ was applied for variant annotation against a database of RefSeq transcripts. A bespoke script was used to assign individual variants as: ‘novel’ if they were not previously reported in the dbSNP137 databases,^9^ 1000 Genomes Project,^10^ the Exome Variant Server (EVS) of European Americans of the NHLI-ESP project with 6500 exomes (http://evs.gs.washington.edu/EVS/),^11^ in 46 unrelated human subjects sequenced by Complete Genomics^12^ or in the Southampton database of reference exomes. Resultant variant files for each individual were subjected to further in-house quality control tests to detect DNA sample contamination and ensure sex concordance by assessing autosomal and X chromosome heterozygosity.^13^ Variant sharing between the two siblings was assessed to confirm sample relationships, and VerifyBamID^14^ was used to check for sample contamination. Sample provenance was confirmed by independent genotyping of a validated SNP panel, developed specifically for exome data.^15^ CNVs across the Xq22.3–Xq23 region were assessed using the R package ExomeDepth.^16^

### Gene selection

Despite no mutations identified by Sanger sequencing, *OCRL1* was reassessed using an alternative sequencing resource. The segregation pattern in the family was strongly suggestive of X-linked recessive inheritance; both maternal half-siblings were affected despite a healthy mother, which suggested they were hemizygous for the mutant allele. The genomic analysis was, therefore, extended to extract all variations on the X chromosome common to the half-siblings. Additionally, we screened for rare, damaging variants across Xq22.3–Xq23 as well as a panel of known genes associated with idiopathic hypercalciuria/nephrocalcinosis (see online supplementary methods).

10.1136/jmedgenet-2016-104100.supp1 supplementary data

### Site-directed mutagenesis

PCMV6-AC-GFP-tagged AMMECR1 vector was obtained from Origene (RG212378). Site-directed mutagenesis was carried out to achieve the G>A point mutation within position 177 in exon 2 of variant 1 of AMMECR1 to confer substitution of amino acid glycine with aspartate. Primers were designed containing the G>A point mutation: forward primer: CGTGGATGCATAG**A**TACTTTTTCTGCC; reverse primer: GGCAGAAAAAGTA**T**CTATGCATCCACG. PCR mutagenesis was carried out using Pfu Ultra DNA polymerase (Agilent Technologies, Stockport, UK) and the following thermo cycle: 2 min at 95°C, (12× cycles of 1 min at 95°C, 1 min at 55°C, 7.5 min at 68°C), 10 min at 68°C.^17^ DNA was digested with Dpn1 for 1 hour at 37°C to cleave the parental DNA template. The mutated vector was sequenced to confirm the *AMMECR1* mutation (Source BioScience).

### Cell culture

HEK293, COS-7 and HeLa cells were plated on 22 mm sterile coverslips in six-well plates in dulbecco modified eagle medium (DMEM)/10% fetal calf serum (FCS). Cells in each well were transiently transfected with 3 μg of either wild-type or mutant GFP-tagged *AMMECR1* using jetPEI transfection reagent, according to the supplier's instructions (Polyplus, Source BioScience, Nottingham, UK). Cells transfected with jetPEI reagent in the absence of DNA were used as a negative control. DNA–PEI complexes were removed from cells after 4 hours and replaced with fresh DMEM/10% FCS/1% Pen/strep.

### Microscopy

Twenty-four hours after transient transfection, cells were fixed with 4% (w/v) formaldehyde in phosphate buffered saline (PBS) for 7 min at room temperature and mounted in VectaShield mounting medium containing the nuclear stain, 4’,6-diamidino-2-phenylindole (DAPI) (VectorLabs). Coverslips were mounted and visualised using a fluorescent microscope (Zeiss).

## Results

### Quality control analysis

Exome data were high quality, evidenced by 80% mapped coverage at a read depth of >20. For both samples, the average depth of coverage was ≥60, online supplementary table 1. The two samples from the affected siblings selected for exome analysis exhibited expected variant sharing for second-degree relatives and no excess of sharing with any other sample on the same despatch DNA plate. Autosomal and X-chromosome heterozygosity were consistent with gender. VerifyBamID^14^ did not indicate any presence of contamination, and the application of a SNP tracking panel^15^ confirmed sample provenance.

### Targeted gene analysis

No coding variation was found in *OCRL1* consistent with previous Sanger sequencing results. Therefore, the analysis was extended to include all coding mutations across the X chromosomes of the two brothers and three novel non-synonymous mutations satisfied the filtering criteria (as outlined in table 1): *AMMECR1* (c.G530A; p.G177D), *KDM5C* (c.4082A; p.R1361K) and *MAGEC1* (c.G919A; p.V307M). The *MAGEC1* (p.V307M) variant was poorly conserved (PhyloP^18^ score of 0.868789) and was not predicted to be damaging and thus removed from downstream analysis. Although *KDM5C* has been associated with Claes-Jensen Type Intellectual Disability (MIM: 314690), which is a syndrome that has been characterised by severe mental retardation, microcephaly and large feet, we removed the novel variant (p.R1361K) from the downstream analysis because it was not predicted to be damaging by the in silico tools CADD^19^ and Polyphen2^20^ (score of 1.2 and 0.033, respectively) Furthermore, *KDM5C* has been associated with mental retardation, whereas we describe a mild developmental delay inconsistent with the severity and nature of the phenotype of Claes-Jensen. The novel non-synonymous variant of unknown significance in *AMMECR1* (c.G530A; p.G177D) warranted further scrutiny with high prediction scores of pathogenicity (Phylop, Polyphen2, CADD and GERP++ scored this variant 0.998909, 1, 5.5 and 5.27, respectively). Moreover, *AMMECR1* has previously been deleted in patients with Alport syndrome, mental retardation, midface hypoplasia and elliptocytosis.^1^ ^21^ ^22^ No rare variants were identified in known hypercalciuria genes (see online supplementary results 1), although *CLDN14* and *PTH* had suboptimal gene coverage by the Agilent V5 capture kit (45% and 86%, respectively). All other hypercalciuria genes had >90% coverage.

**Table 1:** Three novel non-synonymous mutations that satisfied the filtering criteria across the 323 variants shared on the X chromosomes between the two half-brothers

Chromosome	bp position (hg19)	Gene	Variant type	Variant info	Phylop^+^	Polyphen	CADD	Gerp^++^
X	109507771	AMMECR1	ns	AMMECR1:NM_015365:exon2:c.G530A:p.G177D	0.998909	1	5.776826	5.27
X	53221984	KDM5C	ns	KDM5C:NM_001146702:exon24:c.G4082A:p.R1361K		0.033	1.26541	
X	140994109	MAGEC1	ns	MAGEC1:NM_005462:exon4:c.G919A:p.V307M	0.868789	0.512688		0.157

A total of 18 049 variants were shared between the half-siblings. The filtering strategy applied was limited to shared variants on the X chromosome only (323 annotated). 318 variants were removed due to their occurrence within the Southampton control cohort of exomes (n=156) regardless of annotated zygosity. Of the five remaining variants, one was removed due to its synonymous annotation, and another was disregarded due to its low MaxEnt splicing score (<3). Three novel (absent from ExAC server, dbSNP and the Southampton in-house control database) non-synonymous variants satisfied the full filtering criteria. Ns, non-synonymous; sp, splicing.

10.1136/jmedgenet-2016-104100.supp2 supplementary data

### Mutation within *AMMECR1*

The *AMMECR1* mutation segregating in our family causes a G>A change at genomic position 530, leading to a glycine to aspartate change at codon 177 in exon 2. *AMMECR1* encodes a protein with a nuclear location whose function is still unknown. The C-terminal region of *AMMECR1* (from amino acid residue 122 to 333) is well conserved, and homologues appear in species ranging from bacteria and archaea to eukaryotes. Exon 2 of *AMMECR1* encodes a domain consisting of six amino acids identically conserved throughout the course of evolution. The putative nuclear localisation and the presence of a glycine-rich N terminus raise the possibility that *AMMECR1* has a regulatory role.

No CNVs or rare variants were observed in either of the affected brothers within the Xq22.3–Xq.23 region (average read depth of 49.66). We confirmed the *AMMECR1* mutation using traditional Sanger techniques and demonstrated hemizygous status in the two half-brothers and heterozygous carrier status in the unaffected mother. The probands' maternal aunt did not carry the *AMMECR1* mutation.

### Elliptocytosis

With evidence to support an association between *AMMECR1* and elliptocytosis, blood films were requested on the brothers at ages 10 and 4, and both smears were reported blindly. The younger brother, proband II(2), had a normal blood film with no evidence of elliptocytosis, but the older brother, proband II(1), had an abnormal film with scattered elliptocytes and anisocytosis. A repeat blood film on proband II(1) 10 months later confirmed the presence of elliptocytes, though the findings were subtle.

### Functional analysis

**Figure 2:**
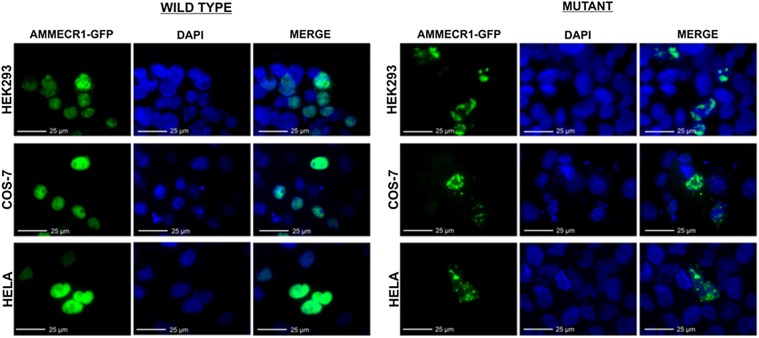
G>A point mutation within position 177 of AMMECR1 gene alters the nuclear expression pattern of the protein. Representative images of HEK293, COS-7 and HeLa cells 24 hours after transfection with a vector containing wild-type or mutant GFP-tagged AMMECR1. Nuclear localisation of AMMECR1 was observed in both wild type and mutant; however, the non-uniform expression pattern of AMMECR1 was only found in cells transfected with mutant AMMECR1.

Analysis of AMMECR1 protein was assessed by GFP expression in transfected cell lines. Colocalisation of GFP with the nuclear DAPI stain was observed in both mutant and wild-type transfected cells, showing localisation of AMMECR1 protein within the nucleus (figure 2). The expression pattern of wild-type AMMECR1 was spherical, overlaying the DAPI staining completely. Mutant AMMECR1 expression was distinct from wild type in all three cell lines tested, with HEK293, COS-7 and HeLa cells exhibiting non-uniform GFP expression within the nucleus. The number of GFP-positive cells was also reduced following transfection with mutant AMMECR1 compared with the wild type. These data confirm that the G>A point mutation within position 177 of exon 2 of *AMMECR1* affects the expression pattern of this protein within the nucleus.

## Discussion

We have identified a single base substitution (G>A) causing a missense mutation in which a non-polar glycine amino acid residue is replaced with a negatively charged aspartate in the AMMECR1 protein. The observed variant codes for a highly conserved residue of AMMECR1 within a highly conserved six-amino-acid motif (LRGCIG). The variant appears novel to this pedigree not occurring in our database of local exomes nor in the publicly available variant repositories.

Functional analysis shows that the G>A point mutation within amino acid 177 of the AMMECR1 protein affects nuclear localisation. Proteasome complexes found within the cytoplasm and nucleus are responsible for targeted degradation of mal-folded or damaged proteins occurring due to missense or non-sense mutations, as well as denaturation and biosynthetic errors.^23^ ^24^ The expression pattern of mutated AMMECR1 we observed within the nucleus is consistent with the altered protein being targeted for degradation by this system. The localisation pattern of the mutant AMMECR1-GFP protein has similarity with the localisation of subunits of the 20S nuclear proteasome, previously described by Baldin *et al*.^25^ The mutated AMMECR1 protein may therefore be targeted for degradation within the nucleus. Degradation of AMMECR1 is also consistent with reduced GFP expression observed in cells transfected with mutant AMMECR1 compared with wild type. Disease states caused by proteolytic degradation of mal-folded proteins as a result of missense mutations include cystic fibrosis, where targeted degradation of the mutant form of the cystic fibrosis transmembrane conductance regulator (CFTR) protein, caused by a missense mutation, prevents normal functioning.^24^

Literature describing *AMMECR1* is sparse; its description and name (Alport syndrome, Mental retardation, Midface hypoplasia and Elliptocytosis Chromosomal Region gene 1) is derived from its cytogenic location within Xq22.3–Xq23 and its previous association with a very rare contiguous gene deletion syndrome originally named Alport syndrome, mental retardation, midface hypoplasia and elliptocytosis (AMME). Jonsson *et al*^1^ first described AMME caused by a deletion including *COL4A5* (Xq22.3) extending proximally to include *AMMECR1*.

**Figure 3:**
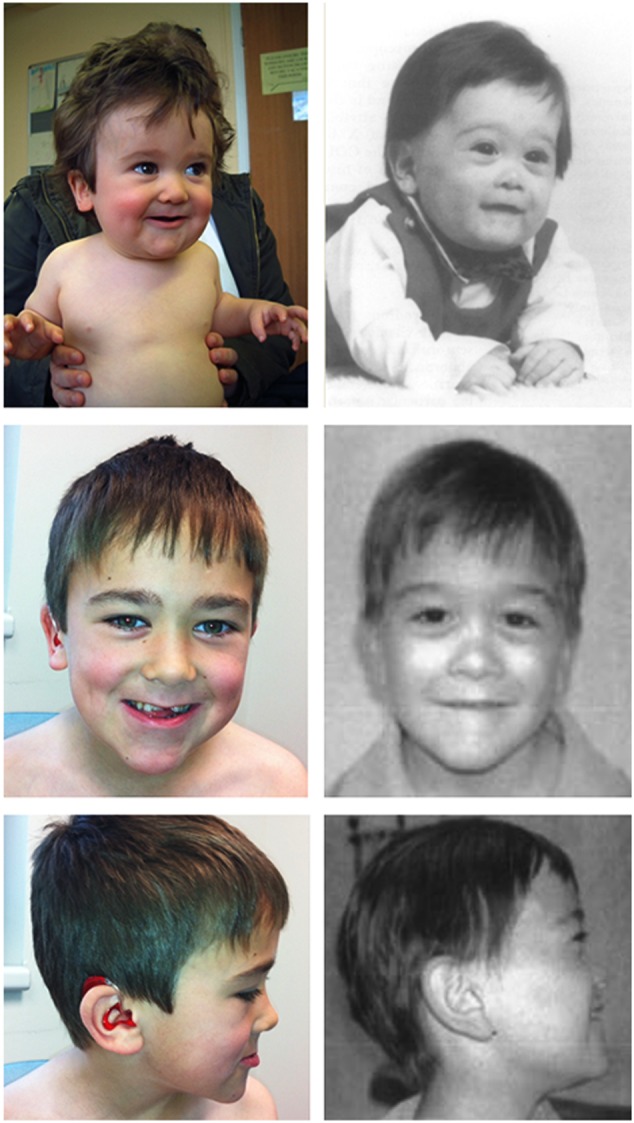
Colour photographs of proband II(1) (left) taken at 11 months and 9 years compared with black and white photos (right) of the patient described by Jonsson *et al* with features of X-linked AMME, taken at 6 months and 12 years. The facial features shared between our study and that of Jonsson *et al* are remarkably striking; shared facial dysmorphology includes a hypoplastic and flat midface, short neck, thin upper lip and a small jaw.

**Figure 4:**
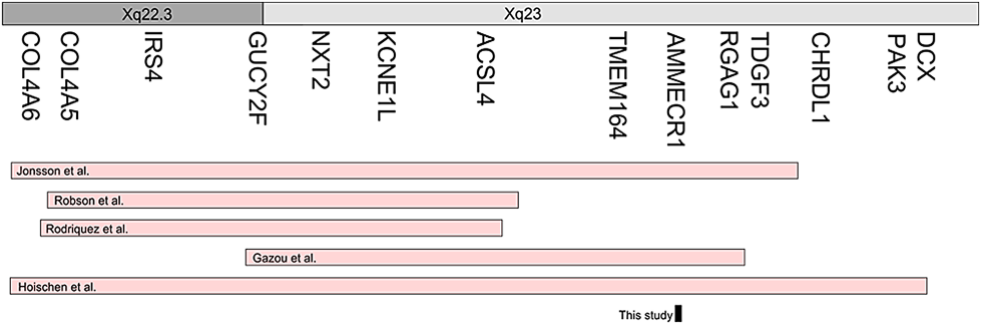
A comparison of deletions across Xq22.3–23 (pink) that have been associated with AMME in five separate reports, in addition to the point mutation in AMMECR1 from this study (black).

We found a striking resemblance between proband II(1) harbouring a single point mutation in *AMMECR1* and the patient originally reported by Jonsson *et al* (figure 3). Contiguous gene deletions of varying sizes within Xq22.3–Xq23 manifest in phenotypes that partially or completely overlap the AMME disease spectrum have been described in five previous reports of which three were inclusive of the *AMMECR1* gene (figure 4). Most of these deletions involve *COL4A5*, the major cause of Alport syndrome, yet mutations in *COL4A5* do not account for the haematological, dysmorphic and developmental characteristics described in AMME. Therefore, it was surmised that these extra-renal abnormalities were attributable to disruption of genes adjacent to *COL4A5*, describing a new contiguous gene deletion syndrome. Subsequently, there has been speculation regarding the roles of genes in this region; however, for the most part their biological significance has been unresolved. Although our report is based on a single family and we must be tentative in drawing firm conclusions without further supportive evidence, the variant is novel. That said, mutant-specific functional evidence and the strong phenotypic overlap between the dysmorphology observed in this study and the original AMME case by Jonsson *et al* advance our understanding of the phenotypic/genotypic overlap between a single point mutation in *AMMECR1* and this contiguous gene deletion syndrome.

Although the single point mutation we observe has clear overlapping features of AMME with previously published cases, this is in the absence of any Alport syndrome. The renal manifestations we describe are clinically disparate from Alport syndrome, consistent with an absence of any *COL4A5* mutations. However, we are mindful that exome data cannot resolve (not uncommon) deep intronic variants in COL4A5, and this is a limitation of our study. As expected, haematuria is only observed in Xq22.3–Xq23 regional deletions that overlap *COL4A5*. Contrastingly, sensorineural hearing loss is consistently observed across all case studies, regardless of direct *COL4A5* involvement. Proband II(1) in our study has hearing loss, as does the proband reported by Gazou *et al*^26^ who harboured a deletion not involving *COL4A5* but inclusive of the *AMMECR1* gene. This may suggest that *AMMECR1* is pathogenically implicated in hearing loss, either alone, or as a modifier gene to *COL4A5* due to their direct protein–protein interaction (see online supplementary figure 1). The sensorineural hearing loss in proband II(1) presented at age 3, earlier than is typical in X-linked Alport syndrome and subsequently progressed. His half-brother, at aged 4, has air conduction values on the cusp of abnormality. His audiogram shows deterioration from one previously, suggesting a progressive pattern of hearing loss without congenital onset.

10.1136/jmedgenet-2016-104100.supp3 supplementary figure Known and predicted AMMECR1 protein-protein interactions (taken from STRING database).

Intellectual disability is prevalent among individuals with deletions across the Xq22.3-23 region; however, the degree is varied. A critical region was identified by Meloni *et al*,^27^ who identified four genes of interest (*ACSL4*, *KCNE1L*, *NXT2* and *GUCY2F*). *ACSL4* has since been implicated in non-specific X-linked mental retardation (MIM: 300387), favouring this gene as the cause of intellectual disability in Xq22.3–Xq23 deletions.^26^ *ACSL4* is deleted in all the microdeletion reports (figure 4). Gazou *et al* have stated that “Deletion of *ACSL4* is probably the major, and possibly only, causative factor for intellectual disability (ID)”…. The moderate degree of ID is similar to patients with point mutations in *ACSL4* and does therefore not suggest additive effects by other genes. Absent or delayed speech is the predominant phenotype in individuals reported by Rodriguez *et al*,^22^ Robson *et al*^28^ and Jonsson *et al*^1^ but this was of a greater severity than reported in these half-siblings (table 2). Standardised, quantifiable measures would aid in accurately mapping intellectual disability severity to chromosomal regions. We observed delayed speech, with no concerns by the time the children attended school, although they did have delayed reading and writing ability and there was parental concern regarding slow progress and concentration difficulties. Of course, delayed speech may be attributable to hearing abnormalities or the submucous cleft palates. Both abnormalities were corrected (with hearing aids and cleft palate repair), which may explain the improvement in speech and language development. Other reports^1^ ^22^ ^26^ ^28^ include generalised hypotonia, contributing to developmental delay. Proband II(1) had significant infantile hypotonia^1^ and delay in gross motor skills in addition. This may be in part secondary to joint hypermobility, which persisted through childhood. As AMMECR1 is associated in the published literature with ACSL4^29^ (see online supplementary figure 1), we propose that this may explain the speech and language delay and early motor delay observed.

**Table 2:** A comparison of phenotypes reported in the literature involving deletions around the Alport locus (Xq22.3) and extending towards the telomere

Feature	Jonsson *et al*^1^ proband (1)	Jonsson *et al*^1^ proband (2)	Robson *et al*^28^ proband (1)	Robson *et al*^28^ proband (2)	Rodriquez *et al*^22^ proband (1)	Rodriquez *et al*^22^ proband (2)	Gazou *et al*^26^ proband (1)	Hoischen proband (1)	This study II(1)	This study II(2)
Sex	Male	Male	Male	Male	Male	Male	Male	Female	Male	Male
Region involved	*COL4A6* to *TDGF3*	*COL4A6* to *TDGF3*	*COL4A5* to *ACSL4*	*COL4A5* to *ACSL4*	*COL4A5* to *ACSL4*	*COL4A5* to *ACSL4*	*GUCY2F* to *RGAG1*	*COL4A6* to *DCX*	*AMMECR1*	*AMMECR1*
Short stature	5th centile	5th centile	−	−	5th centile	−	−	N/A	0.4th centile	<0.4th centile
Infantile hypotonia	+	+	+	+	−	−	−	N/A	+	-
Hypermobility	+	+	−	−	−	−	−	+	+	+
Hearing loss	Mixed	Sensorineural	Sensorineural (normal hearing at 2 years)	Sensorineural	−	−	Sensorineural	Sensorineural	Mixed (normal hearing until 3 years)	Conductive
Submucous cleft palate and bifid uvula	−	−	−	−	−	−	−	−	+	+
Haematuria	+	+	+	+	+	+	−	+	−	−
Nephrocalcinosis	−	−	−	−	−	−	−	−	+	+
Flattened nasal bridge	+	+	+	+	+	+	+	+	+	+
Midface hypoplasia	+	+	+	+	+	+	−	+	+	+
Digital abnormalities	Persistent fetal pads, increased space between index and middle fingers as well as first and second toes, second toe clinodactyly	Persistent fetal pads, increased space between index and middle fingers as well as first and second toes, second toe clinodactyly	Persistent fetal pads, metaphyseal dysostosis	Persistent fetal pads, metaphyseal dysostosis	−	−	Bilateral syndactyly of second and third toes and clinodactyly of second toes	Small hands and fingers, laxity of finger joints, flatfeet, bilateral sandal gaps	Square hands and fifth finger clinodactyly	Square hands and fifth finger clinodactyly
Ocular abnormalities	Myopia	Microstrabismus	−	−	Hyperopia and astigmatism	Hyperopia	–	–	–	Cataract, convergent squint
Speech and language delay	Receptive and expressive speech delay (measured at 5 years and 9 months, predicted age 2–3 and 1 years, respectively)	Developmental delay (less severe phenotype compared with brother (1))	MR (measured at 8 years, patient functioning at the level of 5 years)	MR	Non-verbal at age 5Some sounds	Significant delay at age 2	Started speech at age 3 years and 6 months	MR (measured at 4 years, patient functioning at level of 2 years)	Early delay, normal at age 4	No words at 2 years, normal at age 5
Delayed reading and writing ability	+	+	+	+	+	+	+	+	+	+
Elliptocytosis	+	+	−	−	−	−	−	−	−	−
Cardiac abnormalities	RBBB, PDA, non-stenotic bicuspid aortic valve, mild LV dilatation	Mild mitral and tricuspid regurgitation	−	−	−	−	−	−	Trivial tricuspid regurgitation, patent foremen ovale	−
Other	Umbilical and inguinal hernia	Umbilical and inguinal hernia	Craniopharyngioma	Craniopharyngioma	Mild motor delay, walked at 17 months, temper tantrums	Mild motor delay, walked at 17 months, temper tantrums	Pyloric stenosis, dental delay, motor delay (walked at 24 months)	Therapy-resistant epilepsy, moderate motor retardation (unsupported sitting at 9 months; unsupported walking at 18 months), subcortical heterotropia	Dental delay with first teeth at 21 months, mild–moderate gross motor delay with developmental catch up	N/A

+, phenotype present; −, phenotype absent; LV, left ventricular; MR, mental retardation; N/A, missing phenotype data; PDA, patent ductus arteriosus; RBBB, right bundle branch block.

Midface hypoplasia is present in all but one (Gazou *et al*) case studies involving the AMME locus.^26^ While no other reports include cleft palate, midface hypoplasia is often observed in individuals with this congenital abnormality. The midface hypoplasia may represent a minimal expression of this phenotype. The dysmorphology reported is variable and includes anteverted nares, downslanting palpebral fissures, peripalpebral fullness, fetal pads, deep-set eyes, wide nasal bridge, broad nasal tip, long philtrum, thin upper lip, downturned mouth, widely spaced teeth, supernumery teeth, facial hypotonia, second toe syndactyly and clinodactyly. Of note, as with our study, there are reports that the facial dysmorphism lessens in severity over time.^21^

**Figure 5:**
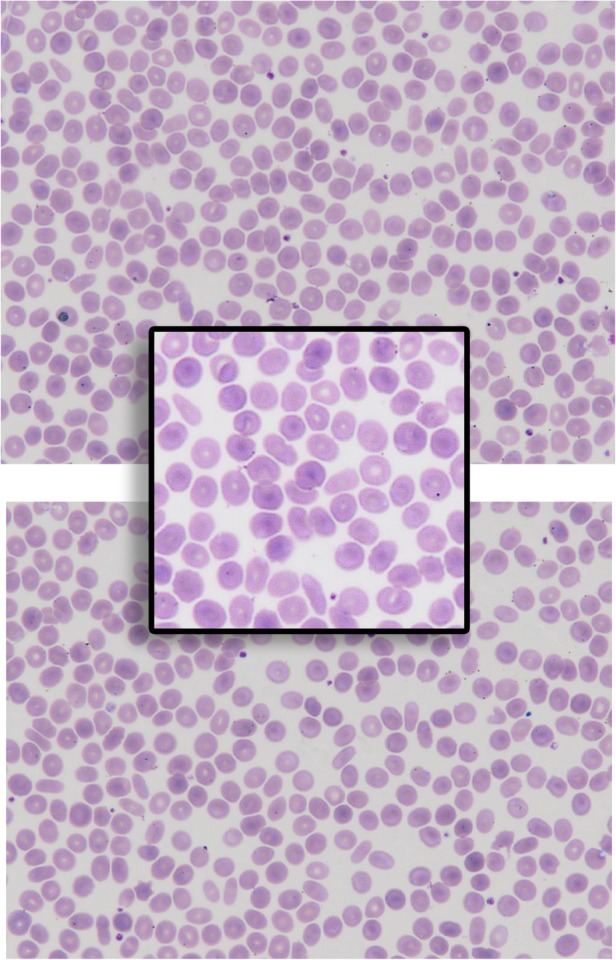
A peripheral blood film (two fields of view and one closer image) of proband II(1) reported as ‘elliptical cells seen’.

Within the AMME locus, elliptocytosis only occurs in regions inclusive of *AMMECR1*, although its penetrance is varied; elliptocytes were observed only in the blood films of the elder brother at age 10 and not in the younger sibling at age 4. Haematological abnormalities were also absent from the probands reported by Gazou *et al*^26^ and Hoischen *et al*^30^ (although the Hoischen proband was female with a uniallelic deletion). It is noteworthy, however, that the elliptocytosis is a mild phenotype (figure 5). Scattered elliptocytes were present and reported on both blood films; however, their subtlety may not warrant an obvious elliptocytosis diagnosis; that said, we report a similar scattering of elliptotic cells consistent with the (mild to moderate) ‘elliptocytosis’ originally reported by Jonsson *et al*.^1^ Absence of elliptocytosis in other studies with microdeletions spanning *AMMECR1* may be due to laboratory under-reporting, particularly if the red cell morphology is subtle. Moreover, aberrant red cell morphology was absent in the younger half-sibling from this study, which may instead reflect variable penetrance or demonstrate a delay in phenotype onset; however, elliptocytosis was detected at 15 months in one of the affected brothers from the Jonsson *et al* study, where the entire *AMMECR1* gene was deleted.

The single point mutation in *AMMECR1* in this study accounts for early speech and language delay, hypotonia, midface hypoplasia and elliptocytosis, and is found in association with nephrocalcinosis, cleft palate, bifid uvula, hearing loss, hypercalciuria, strabismus, cataracts, hypermobility, hypotonia and delay in eruption of primary dentition; although the phenotypic penetrance is varied. The strong interactions between AMMECR1 and neighbouring proteins of the AMME locus render genotype/phenotype stratification difficult. Nephrocalcinosis, hypercalciuria, cataracts (in proband II(2)), submucous cleft palate and bifid uvula are unique to our study and may be entirely incidental; we do not have sufficient evidence in support of any causal relationship between *AMMECR1* and these manifestations. It is possible, though unlikely, that the half-brothers may have a second (unrelated) condition that explains their additional features. We excluded known genetic causes for nephrocalcinosis and idiopathic hypercalciuria in 12 of 14 genes assessed. Two genes, *PTH* and *CLDN14*, had suboptimal gene coverage; and although no variants were found, we are cautious to exclude them. However, we are reassured that both *CLDN14* and *PTH* are not on the X chromosome.

## Conclusions

In this study, we have applied contemporary sequencing technology and unbiased filtering to a half-brother pair and identified a single point mutation within an X-linked gene that causes a severe phenotype, previously described as part of a contiguous gene deletion syndrome. Functional analysis shows that the novel mutation in *AMMECR1* affects protein localisation, possibly due to degradation of the mal-folded protein. We propose that *AMMECR1* has a critical role in the extrarenal manifestations of AMME, either alone or due to its predicted protein–protein interactions with other genes in the AMME locus. Our study narrows the AMME locus to a single point mutation in *AMMECR1* that appears critical in the pathogenesis of midface hypoplasia and elliptocytosis, and contributes to early speech and language delay, hypotonia and hearing loss, and may, in addition, play a role in dysmorphism, nephrocalcinosis and submucous cleft palate.
