# Supplementary material for: AMMECR1: a single point mutation causes developmental delay, midface hypoplasia and elliptocytosis
Source: J Med Genet. 2016 Nov 3;54(4):269–77. doi: 10.1136/jmedgenet-2016-104100 (PMC5502304; doi:10.1136/jmedgenet-2016-104100)
Supplement: supplementary data [file jmedgenet-2016-104100supp001.pdf]

Table 1 | Summary statistics for exome sequencing - mapping and coverage

ID	Variants	% Autosome heterozygosity	% X heterozygosity	Pipeline gender
**II(1)**	24934	61.358	18.49	male
**II(2)**	25001	61.2082	18.13	male

Variants – total called variants; % Het – genome wide % of calls heterozygous; % X Het - % calls mapped to X chromosome heterozygous; Pipeline. Gender – apparent gender based upon % X Het.
