# Supplementary material for: AMMECR1: a single point mutation causes developmental delay, midface hypoplasia and elliptocytosis
Source: J Med Genet. 2016 Nov 3;54(4):269–77. doi: 10.1136/jmedgenet-2016-104100 (PMC5502304; doi:10.1136/jmedgenet-2016-104100)
Supplement: supplementary data [file jmedgenet-2016-104100supp002.pdf]

Supplementary table 2

II(1)	II(2)	Chr	Zygosity	gene	var	var info	Phylop	Gerp	rsID	cg46	1000g	ESP	Soton DB	Soton NDDB	PHRED
X	X	7	HOM	ATP6V0A4	SNV	NM_130840:exon16:c.T1812C:p.H604H	.	.	rs3807154	0.62	0.7	0.719186	4,1,5	313	222
X	X	7	HOM	ATP6V0A4	SNV	NM_130840:exon15:c.C1662T:p.F554F	.	.	rs1026435	0.696	0.73	0.721512	4,1,5	317	222
X	X	7	HOM	ATP6V0A4	SNV	NM_130840:exon2:c.T5C:p.V2A	0.781742	1.58	rs10258719	0.674	0.66	0.725116	1,3,4	317	222
.	X	7	HOM	ATP6V0A4	splicing	NM_130841:exon4:c.196+10->TTTTTTTTTT	.	.	.	.	.	.	0,0,0	13	114
X	X	3	HOM	CASR	SNV	NM_000388:exon7:c.G2244C:p.P748P	.	.	rs2036400	0.957	0.97	0.999418	6,0,6	340	222
X	X	3	HOM	CASR	nsSNV	CASR:NM_000388:exon7:c.G3031C:p.E1011Q	0.983708	5.36	rs1801726	0.913	0.92	0.959535	6,0,6	339	222
.	X	21	HET	CLDN14	SNV	NM_012130:exon2:c.G687A:p.T229T	.	.	rs219780	0.12	0.15	0.210116	0,2,2	101	67
.	X	21	HET	CLDN14	SNV	NM_012130:exon2:c.C243T:p.R81R	.	.	rs219779	0.228	0.2	0.257326	0,4,4	134	220
X	.	3	HET	CLDN16	nsSNV	NM_006580:exon2:c.G379A:p.G127R	0.999683	5.37	.	.	.	.	0,0,0	1	198
.	X	3	HET	CLDN16	splicing	NM_006580:exon1:c.324+10T>C	.	.	rs1491994	0.109	0.14	0.248023	2,0,2	122	98
.	X	3	HET	CLDN16	fs_del	NM_006580:exon1:c.166delG:p.A56fs	.	.	.	.	.	.	2,0,2	120	217
X	X	12	HOM	VDR	SNV	NM_001017536:exon10:c.T1206C:p.I402I	.	.	rs731236	0.228	0.26	0.394419	0,1,1	202	205
X	X	12	HET	VDR	nsSNV	NM_001017536:exon3:c.T152C:p.M51T	0.978818	3.23	rs2228570	0.728	0.65	0.61407	2,4,6	278	70

Gene	% Coverage
CASR	93.81
PTH	86.49
CLCN5	91.95
TRPV5	100.00
CLDN14	44.98
CLDN16	97.22
PMCA4	95.15
VDR	96.85
OPN	91.68
MGP	100.00
AE1	97.31
ATP6V1B1	100.00
ATP6V0A4	90.35
SLC34A1	98.35
HPRT1	100.00

A table filtered for all variants found in known hypercalciuria genes. Variants common to the half-siblings II(1) and II(2) are highlighted in green. All shared variants were common in population databases and in the local Southampton non-disease database (Soton NDDB) and are highlighted in orange. Percentage gene coverage for all hypercalciuria genes are tabulated and suboptimal coverage (<90%) is highlighted in red.

Chr – chromosome; var – variant type; SNV – synonymous single nucleotide variant; nsSNV – non-synonymous single nucleotide variant; var info – variant info; cg46 – complete genomics 46 database; 1000g – 1000 genomes population database; ESP – exome sequencing project population database; Soton DB – Southampton disease database; Soton NDDB – Southampton non-disease database.
