# Supplementary material for: AMMECR1: a single point mutation causes developmental delay, midface hypoplasia and elliptocytosis
Source: J Med Genet. 2016 Nov 3;54(4):269–77. doi: 10.1136/jmedgenet-2016-104100 (PMC5502304; doi:10.1136/jmedgenet-2016-104100)

Supplementary Figure 1

Known and predicted AMMECR1 protein-protein interactions (taken from STRING database).

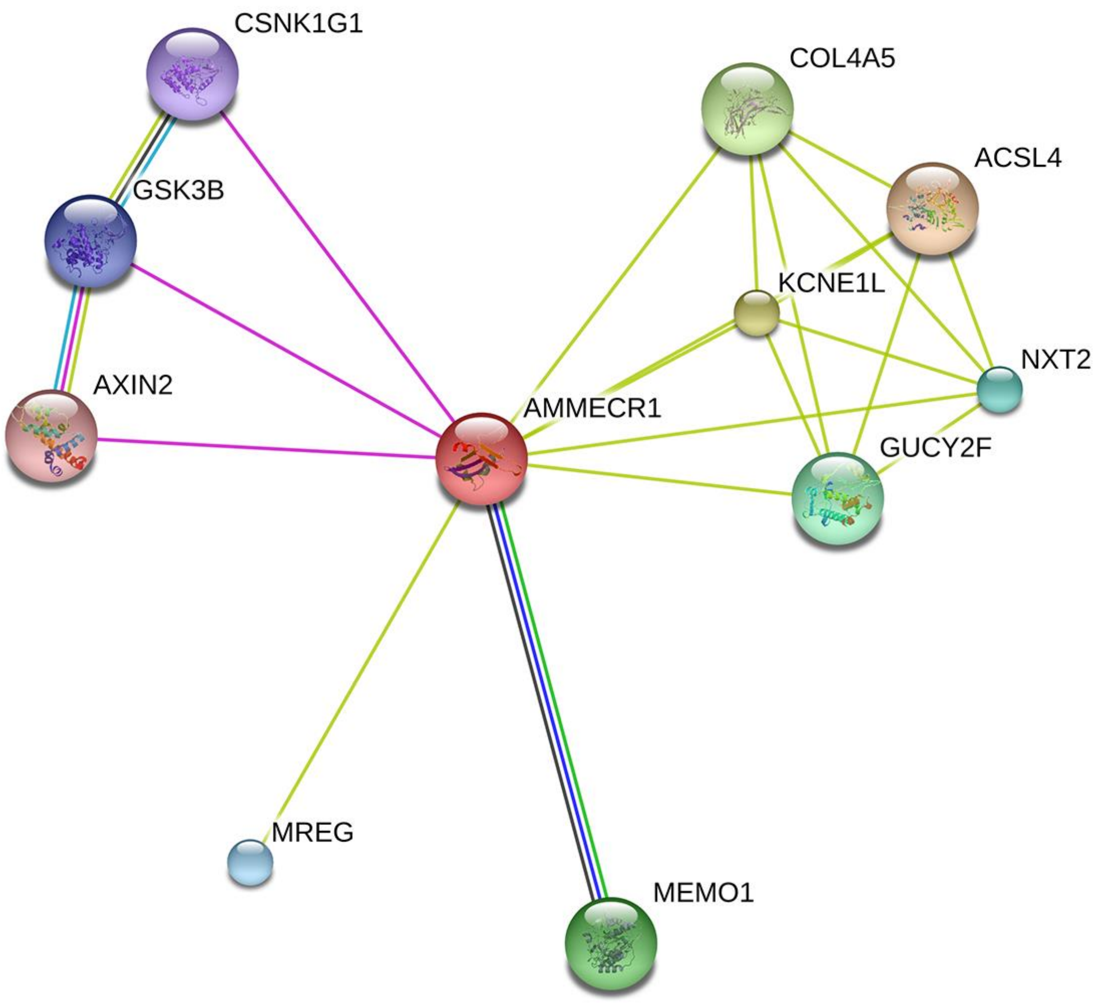

Supplement: supplementary figure — Known and predicted AMMECR1 protein-protein interactions (taken from STRING database). [file jmedgenet-2016-104100supp003.pdf]
